# Hospital and Institutional News

**Published:** 1912-01-06

**Authors:** 


### HOSPITAL AND INSTITUTIONAL NEWS.

### THE NEW YEAR HONOURS.

The third honours list within six months can hardly, perhaps, excite so much public interest as its predecessors, but the hospital world will be glad to note the distinctions that have fallen to two hospital governors, four medical men, and one medical officer of health. Mr. L. E. Ralli, a governor of the London Hospital and a generous supporter of the voluntary system, has been made a baronet. The honour of knighthood has been given to Mr. Charles Behrens, twice Lord Mayor of Manchester, who presided at the great conference of the British Hospitals Association in September last, and has been active in his support of local hospitals. Dr. A. Newsholme, M.D., Medical Officer of the Local Government Board, has been made a Companion of the Bath, which distinction gives official recognition to his well-known services in many branches of vital statistics and preventive medicine.

### THE NEW MEDICAL KNIGHTS.

Dr. John Hawtrey Benson, P.R.C.P. Ireland, is medical adviser in Ireland to the Colonial Office, and Fellow of the Academy of Medicine, Ireland; Dr. Robert John Collie, M.D., C.M., is Chief Medical Officer to the Metropolitan Water Board, and has made a special study of medical jurisprudence in relation to accident legislation; Mr. James Mackenzie Davidson, M.B., C.M., is Consulting Surgeon to the Röntgen ray department of Charing Cross Hospital and has made a special study and written several papers on *x*-ray work; Dr. George Henry Savage, M.D., F.R.C.P., is the Consulting Physician to Guy's Hospital and to Earlswood Idiot Asylum, and formerly Physician-Superintendent of Bethlem Royal Hospital, Examiner in Mental Physiology, University of London, and Lecturer on Mental Diseases at Guy's Hospital. All these gentlemen have received knighthoods, and their distinctions embrace a divergent and fairly representative share of the field of modern medicine.

### NOTIFICATION OF PULMONARY TUBERCULOSIS.

Hospital physicians and resident medical officers, as well as private practitioners, will do well to note that the Order of the Local Government Board making compulsory the notification of pulmonary tuberculosis in England and Wales came into force on January 1, 1912. Ignorance of the law is not accepted as an excuse for failure to comply with it; and although no doubt allowances will be made by those in authority for the unfamiliarity of this new obligation, still it is incumbent upon all members of the medical profession to take note of the Order and act up to it. In hospitals and dispensaries this will entail a good deal of additional work for the medical staff. Since junior practitioners filling resident appointments are often brought into contact with considerable numbers of consumptive patients, it would be well if every hospital in England and Wales were to post a notice in the medical out-patient rooms and casualty rooms calling the attention of the staff to this new Order. A case occurred not very long ago in which a house physician at a large hospital was summoned and fined for failing to notify a case of typhoid fever which he had admitted to his wards. The crime was discovered because the patient died, and his death certificate was the first intimation the authorities had of the occurrence of the case, whereupon they decided upon disciplinary action. A similar result may easily occur in connection with, let us say, a case of hæmoptysis; and since such incidents are annoying to medical officers and may be held to reflect slightly on the hospital, it is worth while for hospital secretaries to take the simple precaution suggested for avoiding them.

### COMPLAINTS OF CHRISTMAS FESTIVITIES.

The reaction after the festivities of Christmas are followed up not infrequently by a scare of some sort. This year the sacred person of Father Christmas himself is being attacked. The grumblers point out that Father Christmas's clothing consists as a rule of cotton wool, and then add the dreadful statement that cotton wool is inflammable. In hospitals, where the reverend father's appearance is usually made in morning or midday light, and where, moreover, electric light is practically the sole illuminant, the risk seems minute. It exists, of course, but then, we fear, risks will continue to exist long after the latest precautions have been taken. In the meantime it would be interesting to know if any serious mishap has ever occurred in hospitals from the inflammable robes of this venerable figure. Where ward decorations have been slowly given up, are Father Christmas's habiliments to follow?

### HOW TO START A FESTIVITIES FUND.

The title of this paragraph is a question that still has to be answered in many hospitals. A practical example, therefore, by an experienced hand is worth recording: "Early in December," writes a provincial hospital secretary, "I initiate a Christmas Festivities Fund, first soliciting contributions from members of the committee, then from others, with the result that I am able annually to obtain about £17 to £20. In addition to this I send out about 150 letters appealing for gifts in kind, which consist of turkeys, cheese, fruit, flowers, evergreens, plants, Christmas tree, tobacco, papers, scrap books, crackers, sweets, cakes, jellies, loan of silver-plate, earthenware, cutlery, etc. The matron and I are assisted by nearly everyone of the salaried staff, who does something towards the additional work at this very busy season. It means very hard work indeed for us all, but we are more than repaid in the knowledge that we can make this season a happy one."

### KINGSTON DOCTORS AND GUARDIANS' DISPUTE.

In our issue of November 25 (page 193) we reported the controversy that has arisen between the Kingston Guardians, the local magistrates, and the local doctors concerning the certification of pauper lunatics. Relieving officers have called in the magistrate, who, as a rule, has decided on the advice of the Poor Law Medical Officer, who thus has received the certification fees, to which the patient's usual medical attendant has felt entitled. A return has now been prepared by the Guardians, showing that out of a total of £155 odd paid in fees £153 has gone into the pockets of one medical officer. The local medical men are expected to appeal to the Local Government Board against the Guardians' regulation that every suspected lunatic should be brought to the workhouse, which the doctors believe to be contrary to the provisions of the Lunacy Act. Dr. Owen Coleman, Surbiton Medical Officer, as we pointed out, was the first to voice the practitioners' complaint, and his fellow district medical officers will eagerly await the result of any appeal that may be taken.

### SERIOUS CHARGES AGAINST NEW YORK HEALTH OFFICERS.

Dr. Doty, Health Officer of the Port of New York, has been called upon to resign, says the *Times* correspondent, the Governor supporting Mr. Bulger's charges that the administration of the State's quarantine "is replete with evidence of gross incompetency." Mr. Bulger was appointed by the Governor to investigate the affairs of the office. It is stated that Dr. Doty's medical confrères have been making strong representations on his behalf. While having refused to resign, Dr. Doty is stated "not to be taking legal proceedings to maintain his office."

### HOSPITAL COMBINATION: A LOCAL EXAMPLE.

Two years ago the Liverpool Council of Voluntary Aid was formed to co-ordinate charitable effort in the city. It won approval outside the place of its origin by forwarding to all members of Parliament copies of the resolutions which were passed at the meetings of the British Hospitals Association in Manchester and London. Its representative on the British Hospitals Association is Mr. H. Wade Deacon, and its first annual report shows that its group committees have been busy at investigation work. In more than one instance the northern hospitals have set the institutional world an example of co-operation and initiative, and the New Year brings home with increased force the paramount necessity outlined by Mr. Alvey in our last issue for combined action on the part of all voluntary hospitals. So far, the British Hospitals Association has provided the only common platform on which the voluntary hospitals have met the threatened attack of the Insurance Act, and we are glad to see how local effort has been succeeded by the combination of the great body of institutions in that association. If other centres showed an initiative equal to that of Liverpool the work of the New Year would be less onerous than it promises to be.

### THE NEW YEAR'S REFERENCE BOOKS.

An up-to-date hospital secretary has to touch modern life at so many points, and to find at once so much information about the personalities of the hour that his shelf of reference books has to grow in scope annually. The old indispensables still hold the field, and, apart from "Burdett's Hospitals and Charities," which is his general encyclopædia, the latest issue of "Who's Who" takes the first place. Messrs. A. C. Black have brought their wonderful volume once more up-to-date. Fry's useful "London Charities," published by Chatto and Windus, is again a satisfactory eighteenpennyworth, and the "Writers' and Artists' Year Book" always commands attention in any hospital where the publication department is kept at a high rate of energy. The "Who's Who Year Book" is a useful summary of many expensive publications and as such carries its own recommendation. Equipped with "Burdett" and these four the secretary's department can start the New Year fully armed with references that in one way or another will be referred to of necessity many hundred times during the coming twelve months.

### REORGANISING A HOSPITAL AT ABERDEEN.

We have been compelled to hold over till this week an account of the crisis which apparently has arrived in the affairs of the Aberdeen Dispensary and Maternity Hospital. The continuation of two types of work in a twin institution does not seem to have worked well: so badly indeed that at present the possibility has been foreshadowed definitely of its extinction. Lord Provost Maitland voices the opinions of a special committee of inquiry which began to sit last March, and which now urges the disjoining of the Dispensary from the Maternity Hospital. More than usually regular annual indebtedness has led this institution to fear for its life, but as the committee of inquiry actually states that the yearly cost of running the separated hospital would be a sum of which more than half can be "obtained from rents and grants," it sounds incredible that the hospital should have more than a healthy difficulty in raising this remaining charge if it is doing its work properly. To appeal to the University for a grant as the Provost suggested may be justifiable, but, on the bare facts of the committee's report only, it will seem to many an extremely lazy way of shirking what a competent management would find no difficulty, especially now that sums equal to half the debt have been promised.

### A CHRISTMAS PROTEST.

It is interesting to note that the Bradford hospitals, in particular the Royal Infirmary and the Eye and Ear Hospital, took advantage of their Christmas celebrations to pronounce a warning as to the probable effects of the Insurance Act. Mr. George Priestman, chairman of the Royal Infirmary, who has already been active, was once again in the field, and his action is worth recording as showing how strong is the feeling which on Christmas Day could still affect the minds of the hospital workers with the heavy forebodings of the New Year. No better proof could be shown as to the sincerity of the hospitals' attitude on this question. Mr. Bernhard Nathan, Dr. Campbell, Dr. Bronner, and Mr. Herbert Jeffery (chairman of the Children's Hospital) were prominent among the speakers.

### AN OLD CONTRIBUTOR'S BEQUEST.

For many years past the West Ham Hospital has regarded the late Mr. Joseph Withers, of Stratford, as one of the oldest, if not quite the oldest, of its subscribers. Staff and patients alike have become familiar with his name from the "Withers' Ward," which was so called after his late wife. The hospital at West Ham has now learned that it is his residuary legatee. This means that the institution will benefit to the extent of £20,000, or, in other words, that approximately one-third of his total estate has fallen to its share. Hospitals need to start this year with reinforcements of courage, and, could the giver realise it, his generous bequest has fallen due to the hospital at a most exceptionally important time. Its chairman, Mr. Thomas Alexander Cook, is to be congratulated at the way in which his hospital is thus able to start 1912.

### THREEPENNY PIECES.

Some two or three years ago an extremely able and sympathetic book dealing with the life of a doctor in a very poor part of South London was published under the title of "Sixpenny Pieces." The allusion was made to the standard fee for advice and medicine which that doctor was accustomed to receive. The book was written from a layman's standpoint; and if, as seemed probable, the doctor was drawn from life, he is one by whose life and works the profession of medicine is highly honoured. Low as the fee just mentioned is, the facts made public at two inquests held at Hackney on December 30 would seem to show that there are practitioners who work for half this sum. The excuse offered to the jury by the doctor who had attended was that he had had no time to visit the two children in question owing to press of other work. He is reported to have said that he attends 80,000 patients a year, a figure beside which the gloomiest forecasts of the effects of the Insurance Act pale into insignificance. It is true that the statements of this practitioner, as reported in the Press, savour of bombastic and exaggerated egotism; and that the two inquests are not in themselves any clear proof of the efficiency of the work done under this system of threepenny fees. But it is also evident that the inhabitants of Hackney are satisfied that they get threepennyworth of value from this doctor, since we have his own statement that "the doorway is crammed and hundreds have to go away." A study of this report may be commended to the members of the National Insurance Medical Association.

### NEW YORK'S LATEST HOSPITAL.

If the daily newspapers are to be believed, New York is soon to possess a hospital twenty-two storeys in height. All things are possible in New York, including mistakes of the first order; but freak hospitals of various kinds have sometimes seemed to be the besetting sin of Americans. Some £200,000, according to rumour, is to be spent on this institution, the building of which, it is stated, has been begun. Hospital architects will not feel that an epoch has been created in their science by the new institution, the maintenance of which, according to the *Telegraph*, will probably be provided by the fees of surgeons engaged in post-graduate study.

### MENTAL HOSPITAL MATTERS AT CARDIFF.

The delayed scheme for an isolation block in connection with Cardiff City Mental Hospital, Whitchurch, has moved one step nearer towards realisation in that the visiting committee is taking the first steps towards estimating its cost. In 1902 an estimate was worked out, but it is now felt that a more modern equipment is necessary and the total cost is expected now to reach £6,000. No doubt the hopes of the hospital's authorities in this respect have been cheered by the recent report of the Lunacy Commissioners, who visited the institution last month. They approved the appointment of Dr. R. V. Stanford as research chemist, and thus gave their countenance to the scientific investigation of the hospital. Their chief recommendation was that steps should be taken to minimise the overcrowding in the men's quarters. As regards the site of the proposed isolation block, Dr. Goodall recommends the north-east corner of the recreation field, which, he says, will not be missed for any purpose.

### ANOTHER HOSPITAL HISTORY.

A really capital attempt has just been made to publish a readable history of the General Hospital, Tunbridge Wells. Thanks to the author, Mr. F. Wadham Elers, who is also treasurer to the institution, and to the attractiveness given by A. G. Pelton, the printer, to such details as type, end-papers, and cover, the "Diary of the Principal Events from 1828 to the Present Time" is one of the most inviting brief hospital histories we have seen yet. The Diary scheme is much more satisfactory than it sounds, and has the merit of giving each year its characteristic share in the hospital's progress. For instance, 1849 is noteworthy as the year in which a hot-water bath first became a permanent fixture, and 1890 as the date of an outbreak of scarlet fever. The illustrations are very numerous and represent the most prominent of those who have been associated with the hospital's work. Mr. F. Wadham Elers is to be congratulated on his undertaking, which itself should make 1912 memorable in those later years the diary of which should be continued on the admirable plan which he has been the first to carry out successfully. Mr. Wadham Elers has been treasurer since 1897.

### CHARGING AN INFIRMARY'S STAFF.

We should hardly have noticed the allegations of neglect recently brought against the Birmingham Guardians, and disproved to the satisfaction of the Board's recent meeting, did they not illustrate two things. The first is how vague are the charges commonly preferred against institutions, and the second is how apt charitable societies are to play the game of vigilance without sifting the evidence on which their dilatory charges are usually based. Those charities whose whole business it is to detect certain classes of cruelty or crime have learnt their lesson, and on the whole, with the onus of legal proof upon them and the necessity of incurring an advocate's fees, are shining examples of carefully substantiated allegation. Other charities, when lured by rumour of injustice to an individual who from his poverty or ineptitude may be qualified for their care, are very apt to rush to conclusions and to rely on hearsay evidence. The chairman at Birmingham, Mr. F. Juckes, said that the receiving of complaints was an experience common to all the guardians, and that the proper course was to take it to a quarter where it could be properly investigated, and then, but not till then, if still unsatisfied, to take further action. The cause of humanity to institutional patients is rarely well served where this is not done.

### DIFFICULTIES IN STARTING COTTAGE HOSPITALS.

The Wells Cottage Hospital has completed the somewhat difficult first year of its life. The wetness of the wards delayed the hospital's occupation till the end of March, and then the floors, after a few months' wear, proved defective enough to require relaying by the contractor. The hospital thus was not able to complete more than thirty weeks of work. Things now have apparently settled down, and the new secretary will start the New Year with the hospital in working order. Mr. H. E. Loynes, who has been secretary for this first difficult period of twelve months, has resigned and becomes the honorary treasurer, his place being filled by Mr. A. Q. Watson, who has naturally been impressed with the responsibilities of his office from the difficulties encountered hitherto. We hope his diffidence will soon give way to confidence, and that the end of 1912 will find Mr. Watson with a balance in hand, notwithstanding the hospital having been at work for every week in the year.

### THE ARBROATH RECONSTRUCTION SCHEME.

So quickly has the appeal on behalf of the reconstruction of Arbroath Infirmary, Dundee, been responded to that, of the £8,000 required, £7,000 has been raised. A special building committee, therefore, has been appointed to adjust the general plans with Dr. Mackintosh, M.V.O., of Glasgow, and with Mr. Garin, the architect. The Committee consists of Provost Alexander, ex-Provost Grant, Mr. Balfour of Inchock, Sir Francis Webster, Mr. A. R. Duncan of Parkhill, and the Hon. F. J. Bruce of Seaton. It is hoped to acquire the present smallpox hospital belonging to the town, which is not well placed for the purpose, and if a new one can be built at Little Cairnie this might be arranged for a consideration. Were this done patients could be received and accommodation for the nurses could be found during the reconstruction of the infirmary.

### KING EDWARD MEMORIAL AT CAMBRIDGE.

It is satisfactory to learn that the Cambridge King Edward VII. Memorial Fund is now at the interesting point when the deliberations of a building committee are in progress. This committee, thanks to local generosity, can look to £11,000 as the amount subscribed since October 1910, and next year the new children's ward and the new out-patient department should be seriously advanced in plan, and probably actually in building. Addenbrooke's Hospital is one of those which is represented every year by an annual report that is not merely informative but readable, and we are glad to take this opportunity of congratulating Mr. Richard F. Coles, the secretary, on the latest addition to the series.

### A MATERNITY HOSPITAL IN DIFFICULTY.

Dr. Thomas Bryce has aptly made use of his recent appointment as one of the Directors of the Glasgow Maternity Hospital to explain its work and importance to the Glasgow public in the hope that its still existing building debt may be paid off. The hospital was removed in 1908, and Dr. Bryce reminds us that "a new building has been required about every twenty years to meet the needs of the charity and the training school." A fact of this kind, so concise a summary of its history, is one that will be remembered, and the eloquent reminder of the double functions of a maternity hospital should excite the sympathy and open the purse-strings of the public. The hospital with its contiguous students' hostel has a very important claim on Glasgow's support.

### THIS WEEK'S DRUG MARKET.

Business in the drug market has been at a standstill during the greater part of the interval that has elapsed since last report appeared, and will not be resumed in earnest for a week or two. Merchants are busy with their annual stocktaking, and until that work is over large purchases are not likely to be made. The tendency to higher prices is still pronounced, and, with the exception of glycerine, the value of which has been reduced, there are practically no changes in a downward direction. The reduction in the price of glycerine was, as mentioned in the last report, expected, and it is by no means improbable that a further decline will take place; the price is still very high, and it would be well to continue to refrain from purchasing for the present. Opium is again dearer, and there appear to be no prospects of a decline in value in the immediate future. Morphine and codeine are also likely to be dearer, notwithstanding the advances announced in the last report. Carbonate of ammonia is dearer. The high value of balsam of tolu is well maintained, and balsam of copaiba is dearer. Chamomiles are tending upward in value. Higher prices are quoted for lithia carbonate and citrate. Oil of cloves tends lower in value. Eucalyptus oil is still inclined towards higher prices, as also is sandalwood oil. Aconite root is dearer. An advance in the quotations for salicylic acid and sodium salicylate is thought probable. The prices of sulphocarbolates have advanced in sympathy with the rise in value of carbolic acid. Buyers of cod-liver oil are still holding off, as good fishing is anticipated in Norway; so much depends on the weather during the coming fishing season that it is not possible to make a reliable forecast, but it might be well to refrain for the present from making purchases of last season's oil.

